# Suitability of nutrients removal from brewery wastewater using a hydroponic technology with *Typha latifolia*

**DOI:** 10.1186/s12896-018-0484-4

**Published:** 2018-11-22

**Authors:** Abebe Gebeyehu, Nurelegne Shebeshe, Helmut Kloos, Solomon Belay

**Affiliations:** 1 Ethiopian Institute of Water Resources, Addis Ababa University, Addis Ababa, Ethiopia; 2 Department of Chemical Engineering, Addis Ababa Science and Technology University, Addis Ababa, Ethiopia; 3 Department of Epidemiology and Biostatistics, University of California, San Francisco, USA; 4 Department of Biotechnology, Addis Ababa Science and Technology University, Addis Ababa, Ethiopia

**Keywords:** Brewery wastewater, Hydroponics, Nutrients removal, Phytoremediation, *Typha latifolia*

## Abstract

**Background:**

This study aims to assess suitability of hydroponic technology for treatment of brewery wastewater in a hydroponic bioreactor using *Typha latifolia*. Triplicated hydroponic bioreactor treatment units were designed, constructed and operated at a hydraulic retention time of 5 days with different surface loadings and mean hydraulic loading rate 0.023 m^3^ m^−2^d^− 1^. Young *T. latifolia* shoots were collected in the vicinity of study site. Wastewater characteristics, plant growth and nutrient accumulation during experiment were analyzed as per APHA standard methods and nutrient removal efficiency was evaluated based on inlet and outlet values.

**Results:**

*T. latifolia* established and grew well in the hydroponics under fluctuations of wastewater loads and showed a good phytoremedial capacity to remove nutrients. Significant removal efficiencies (*p* < 0.05) varied between 54 and 80% for Total Kjeldahl Nitrogen, 42 and 65% for NH_4_^+^ -N, 47 and 58% for NO_3_^−^ -N, and 51 and 70% for PO_4_^3−^-P. The system improved the removal up to 29% compared to control and produced biomass of 0.61–0.86 kg dry weight (DW) m^− 2^. Nutrients retained were up to 21.17 g N kg^− 1^ DW and 2.87 g P kg^− 1^ DW.

**Conclusion:**

The significant nutrients reduction obtained and production of biomass led us to conclude that hydroponics technology using *T. latifolia* has suitability potential for treatment of brewery wastewater and similar agro-industrial wastewaters. Thus it could be considered as a promising eco-friendly option for wastewater treatment to mitigate water pollution. Integration of treatment and production of biomass needs further improvement.

## Background

Water pollution is becoming a major concern of the entire world due to rapid population growth, urbanization, unsuitable and non-affordable treatment technologies and inadequate management. Addis Ababa, the capital of Ethiopia, generates an estimated annual volume of more than 49 million m^3^ wastewater, of which about 4 million m^3^ are industrial wastewater [1]. There are over 2000 registered industries in Addis Ababa (65% of all industries in the country) most of them located along river banks [2]. Major industries contributing to wastewater generation in the city are tanning and leather manufacturing industries, distilleries and breweries, oil mills, dairies and textile, food processing chemical, soft drink, pulp/paper and metal industries. In low-income countries, including Ethiopia, only a small proportion of the wastewater (8%) is being treated [1, 3]. Many industries release their effluents into nearby streams and rivers, which causes ecological upsets and constitutes a public health risk that requires proper industrial waste management.

The Ethiopian brewery industry is a multi- national business complex that has shown enormous increases in beer production and marketing in recent years because of rapid increases in beer consumption (24% per year) [4]. It roughly doubles the average annual growth rate in gross domestic product (GDP) of the country. Breweries generate large volumes of wastewater through a sequence of processes. Brewery wastewater has a high content of nutrients (nitrogen and phosphorus) [5, 6]. Nitrogen primarily comes from malt, adjuncts and nitric acid used for cleaning. Discharge of yeast also contributes to the amount of nitrogen in the wastewater. Phosphorus, which comes from cleaning agents, is usually found in concentrations ranging from 30 to 100 mg l^− 1^ depending on the water ratio and cleaning agents used [5].

The discharge of untreated or partially treated industrial wastewater with high amounts of pollutant loads, including nutrients (nitrogen and phosphorus) into nearby rivers are a source of serious concern for the river banks and riverine communities downstream of Addis Ababa [7]. Increasingly research is aiming at low cost, decentralized and environmental friendly approaches to control pollution from industrial wastewater [8, 9]. Increasing efforts are also being made to develop innovative technologies that can recover and reuse wastewater [3, 6].

Development of alternative treatment methods that utilizes the advantages of natural processes in the ecosystem is increasing in the area of wastewater management [10, 11]. Various studies have shown that hydroponics has been found to remove nutrients more efficiently and ecologically friendly than constructed wetlands and as a wastewater technology requires less area, is inexpensive and can be implemented onsite [11, 12]. It is one of the phytoremediation techniques that attract interest in researches of wastewater treatments.

The removal mechanisms of pollutants from wastewater in a phytoremediation technology involve the combined biological, chemical and physical processes with microbial communities, macrophytes and media employed [13–15]. Nitrogen and phosphorus are among the pollutants of concern in wastewater treatment. In wastewater, nitrogen is present mostly in the form of organic nitrogen (Total Kjeldahl Nitrogen (TKN)), ammonia- nitrogen (NH_4_^+^- N) and nitrate-nitrogen (NO_3_^−^- N).

Phytoremediation technologies such as hydroponic systems employ plants to enhance the mineralization and removal of contaminants from wastewater [16, 17]. This process relies on the life interactions of various species of bacteria, the roots of plants, gravel, sun and water. They all contribute both directly and indirectly in the removal of pollutants from the wastewater. Dipu et al. [18] reported that phytoremediation of diary effluent reduced significantly pollutants using macrophytes like cattail (*Typha sp.), Eichhornia sp., Salvinia sp.* and proved it as a promising technology for dairy effluent control. In addition, they indicated a need for further research in phytoremediation. Other studies described the phytoremedial role of the macrophytes *Phragmites, Canna, and Symphytum officinale L.* in treating food industry wastewaters from olive mills, wineries and aquaculture [19].

The potential use of hydroponics should be researched for agricultural, industrial, horticultural wastewaters as a new approach [20]. The gravel media hydroponics method of wastewater treatment played an important role in removing pollutants from wastewater [21]. Other studies also indicated removal efficiencies ranged from 47 to 91% for nutrients (nitrogen and phosphorus) using hydroponics planted with different plant species [22–24]. In addition to pollutant removal, hydroponics can help in growing biomass for value-added materials or energy which also attracts interest in the agro-processing industries [25, 26]. The use of macrophytes has become widely accepted and is an increasingly common alternative in wastewater treatment [13, 27]. These macrophytes are stable toward climatic changes and in the medium in which they are growing. Among the macrophytes, *T. latifolia* is the most common of all aquatic and wetland plants used for municipal and domestic wastewater treatment [28, 29]. It is a fast growing perennial plant with high biomass production that can establish and propagate easily. Plant has also significant nutrient uptake capacity and a great reproduction potential [30]. *T. latifolia* is locally available and accessible macrophyte, which was collected in wetlands inside the compound of Addis Ababa Science and Technology University where the study was carried out. There are studies on using *T. latifolia* for treatment of industrial wastewater [31]. In addition, microorganisms play a major role in removal of contaminants by transforming and/or accumulating them and convert them into their own biomass. Microorganisms break down inorganic nitrogen mostly by denitrification which converts nitrate to nitrogen gas, which escapes from the wastewater resulting in the removal of NO_3_^−^-N.

For brewery wastewater treatment, detailed research data on the suitability of hydroponic systems planted with *T. latifolia* to treat brewery wastewater is still lacking. Some previous studies in constructed wetlands planted with *T. latifolia* indicated its use for treating industrial wastewater [32, 33]. Therefore, this study aims to assess suitability potential of hydroponics technology planted with *T. lattifolia* as an option for brewery wastewater treatment in an eco-friendly way in Addis Ababa, Ethiopia. The findings of the study may serve as an input in the search for decentralized and environmental-friendly brewery wastewater treatment methods.

## Methods

### Experimental site

The site is located at the premises of Addis Ababa Science and Technology University in Addis Ababa. This university is at an altitude of 2326 m above sea level and is located at 8°58’N 38°47′E. The climate is of the subtropical highland type, with average annual temperature, rainfall and relative humidity of 15.9 °C, 1089 mm and 60.7%, respectively.

The hydroponic treatment system was placed under a greenhouse to provide a protected environment for plant vegetation and other activities for the wastewater treatment processes during the study period. Adequate air circulation under the roof ensured environmental conditions similar to the external environment; ambient air temperatures ranged from 11 to 24 °C and sunlight could effectively penetrate the greenhouse.

### Design and construction of a pilot hydroponic system

The main experimental materials used were *T. latifolia*, fine and medium size gravel from the locality and wastewater sourced from St. George Brewery located in the center of Addis Ababa. The porosity of the gravel medium was determined by the water displacement method by determining void volume and gravel volume using initially known volume of water. The ratio of the void volume (Vvoid) to the volume of the gravel (Vgravel) and void volume give the porosity of the gravel medium. Thus the porosity was calculated by the equation:

1 $$ Porosity(n)={V}_{void}/\left({V}_{Gravel}+{V}_{void}\right) $$

Using eq. (1) the porosity of gravel medium used for the experiment (predominantly medium size) was determined to be 0.39. Locally available young shoots of similar sizes were collected from marshy lands and banks of the nearby Akaki and Fanta rivers and transported to the study site.

**Fig. 1:**
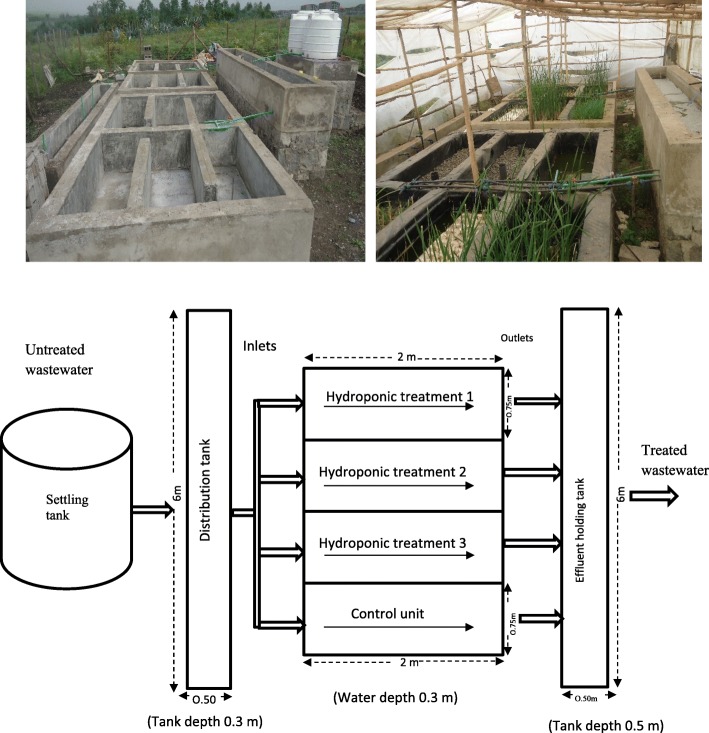
Constructed pilot hydroponic wastewater treatment system inside a green house

The main characteristics and operation parameters of the gravel bed hydroponic treatment system are shown in Table 1. Three hydroponic bioreactor treatment units (HUs) arranged in parallel with one control unit were designed and constructed. In addition, the system has a 1 m^3^ primary settling tank and a 1.5 m^3^ distribution tank before the inlets of the treatment units. The units were made of concrete blocks and fitted with a polyethylene liner to prevent leakage (Fig. 1). Fittings, pipes and valves were used during the installation of the treatment systems.

**Table 1:** Design characteristics and operating parameters of the gravel bed hydroponic treatment units

Design parameters	Values
Number of treatment units	4
Length (L)	2 m
Width (W)	0.75 m
Unit area	1.50 m^2^
Gravel depth	0.40 m
Gross capacity	0.60 m^3^
Macrophyte type	*Typha latifolia*
Operational parameters	
Hydraulic loading rate	2.33 cm d^− 1^
Loading rate	
TKN	0.44–1 g m^−2^d^−1^
NH4+ − N	0.42–0.72 g m^− 2^d^− 1^
NO_3_^−^ - N	0.2–0.4 g m^− 2^d^− 1^
PO_4_^3−^-P	0.19–0.54 g m^−2^d^− 1^
Hydraulic retention time	5 days

The design adopted 5 days of hydraulic retention time (HRT) from experiences of previous studies [10, 29, 34]. Average wastewater depth (h) in the medium for each treatment unit was 0.35 m based on the potential root growth of *T. latifolia* [29, 35] and effective gravel depth of the medium was 0.40 m to ensure subsurface flow of the wastewater during the treatment process (Table 1). The design height of the treatment units was 0.65 m, with 0.25 m increment to serve as a freeboard for plant safety and monitoring.

The required dimensions of the treatment units were calculated based on eq. (2).

2 $$ HRT= nhA/{Q}_{in} $$

where n = porosity of the gravel media, h (m) = effective water depth A (m^2^) is the surface flow area and Q_in_ (m^3^ d^− 1^) is the inflow of wastewater. To maintain plug flow conditions, suitable aspect ratio (length: width) was chosen (2.67: 1) depending on the design intention for this system [29].

The calculated surface flow area was further employed to calculate the hydraulic loading rate (HLR) that provides a measure of the volumetric application of wastewater into the hydroponic treatment unit using eq. (3).

3 $$ HLR={Q}_{in}/A $$

Each treatment unit was provided with inlet and outlet structures for complete wastewater flow within the system. At the bottom of the treatment units a thin layer of overlaying sand was placed to prevent damage of the polyethylene impermeable film from sharp points of the gravel particles when filled in the beds.

### Experimental set up and operation of the hydroponic treatment system

After construction of the hydroponic system, the treatment units were filled with gravel media of sizes ranging from 8 to 25 mm diameter and atop thin layer of sand for plant root support and provision of surface area for microbial attachment sites. Each unit has 0.20 m^3^ void volume (to be filled by the wastewater) with cross sectional area of 0.26 m^2^.

The experiment was arranged in a subsurface horizontal and continuous flow mode in which the flow of wastewater was maintained below the surface of the gravel media. The level of wastewater to be treated in each unit was kept constant. An elbow arrangement installed at the outlets regulated the water level in the bed. Inlet and outlet flow of wastewater were adjusted to maintain the HRT. Two perforated 3.8 cm diameter and 60 cm long pipes were placed inside each reactor unit near the inlet and outlet to measure wastewater depth and also serve as inspection box for wastewater level check and for aeration purposes.

The roots of the collected young plant shoots of *T. latifolia* were washed carefully with tap water to remove adhering soil and sediment prior to use. Then the tops and roots of the selected young and healthy *T. latifolia* were pruned to 20 cm and 10 cm, respectively. The three replicate hydroponic bioreactor treatment units were planted at a density of 16 *T. latifolia* shoots per square meter [36] and there was one control unit without *T. latifolia.* The number of plants at the beginning of the experiment was 24 in each replicated treatment unit. Thus 72 *T. latifolia* were placed in the gravel media at the initial stage of the plantation. After planting, the treatment units were flooded with tap water to about 10 cm above the gravel layer and the plants were left to grow eight weeks to let the system settle to a relatively steady state [32].

Before commencement of the full operation, a serial exposure of raw brewery wastewater feed was introduced into the hydroponic bioreactors. The wastewater was mixed with 75% tap water dilution, gradually increasing wastewater/tap water ratios until only wastewater was added after 3 months. During this acclimatization period, roots of the *T. latifolia* plants in the gravel hydroponic units at a depth of approximately 15 cm below the gravel surface were exposed to the available nutrients as the diluted wastewater flowed slowly through the entire treatment unit. Plants grew rapidly after a few weeks. The survival condition was monitored and dead shoots were replaced after 15 days. When fully operated, each treatment unit was fed with the wastewater by gravity from the settling tank through the distribution system having a flow-adjustable valve fitted in it. To meet the objectives set by the project, the experiment was carried out during 1 year, from January 2015 to January 2016.

### Wastewater sampling and analysis

Raw brewery wastewater was collected in the basins from a manhole to which wastewater from different sections of the brewery were added and channeled to the existing treatment plant. Inlet and outlet wastewater samples were collected on a monthly basis from the hydroponic treatment systems in the study period. For the purpose of characterization and performance evaluation, the following parameters were determined based on standard methods for the examination of water and wastewater [37]: total suspended solids (TSS Dried at 103–105 °C), total dissolved solids (TDS Dried at 180 °C), total Kjeldahl nitrogen (TKN; Kjeldahl Test), nitrate nitrogen (NO_3_- N; ultraviolet spectrophotometric screening methods), ammonia nitrogen (NH_3_ - N; distillation methods); phosphates (PO_4_^3−^, Vanadomolybdophosphoric Acid Colorimetric Method), sulfates (SO_4_^2−^ Turbidimetric Method), biological chemical demand (BOD_5_; 5-days BOD Test), chemical oxygen demand (COD; and the Open Reflex Method). During the entire study period a total of 52 wastewater samples were analyzed for required water quality parameters. Temperatures and pH were measured on-site during sample collection using handheld portable water quality measuring instruments using a digital thermometer (WT-1) and pH-meter CP-105.

### Plant sampling and analysis

At the end of the experiment, above-ground biomass of *Typha* plant samples from each treatment unit 2 at the inlets, 2 at the middle and 2 at the outlet zones were harvested from the gravel surface and transported to the laboratory for analysis. Although, Tanner [38], Brisson and Chazarenc [39] indicated that aboveground and belowground biomass consideration is important for analysis of nutrient removal using phytoremediation, aboveground biomass of *Typha* plant samples was considered. Another study reported higher concentrations of N in aboveground biomass than in belowground biomass in nutrient removal analysis using wetlands [40]. For simplicity, excavation reasons and adequacy for removal comparison, belowground biomass sample of *T. latifolia* was not considered in the present study. Furthermore, aboveground biomass is important in estimating the amount of biomass to be harvested for reuse [41].

The plant samples were oven-dried at 65 °C for 4 days, to constant weight and finely ground and nutrient analyses were carried out for harvested biomass in terms of nitrogen and phosphorus contents at the laboratory as per standard methods. Subsamples of the dried powder were homogenized and the contents of N (Kjldahal technique) and phosphorus (Nitric Acid-Hydrochloric Acid Digestion and phosphorus) were analyzed.

The number of plants and shoots per square meter were counted manually in each unit at the end of the experiment. In addition, aboveground plant growth measurements such as plant height and number of leaves were monitored and recorded on individual stems marked in the center of each experimental unit during the study. The monitoring period lasted one vegetative cycle of 7 months for performance testing.

### Data analysis

Statistics Product and Service Solutions (SPSS Statistics Version 24 package 24) and Microsoft Excel were the statistical tools used for sample data analysis. Comparison of the performance among the hydroponic treatment units for nitrogen and phosphorus removal were analyzed using ANOVA (one-way analysis of variance). Multiple comparison tests between inlet vs control (effect of media alone), HUs vs control (effect of vegetation) and inlets versus HUs (effect of influent) for their nutrient removal were also performed with 95% confidence interval. On monthly basis during the study period, descriptive statistics and percentage removal of the nutrients measured at the inlets and outlets of the HUs and the control unit were used to represent the results of data analysis of the samples. The percentage of removal for each nutrient was calculated to get the treatment efficiency of the system using the following equation:

4 $$ \mathrm{Removal}\ \mathrm{efficiency}\ \left(\%\right)=\left(\raisebox{1ex}{$\left({C}_{in}-{C}_{out}\right)$}\!\left/ \!\raisebox{-1ex}{${C}_{in}$}\right.\right)\ast 100 $$

where C_in_ is inlet concentration and C_out_ is outlet concentration of the nutrients.

## Results

### Wastewater characteristics

Physicochemical characteristics of raw brewery wastewater are presented in Table 2. The result showed that raw brewery wastewater has high levels of organic matter, nutrients and solids, which corroborates the study by Jaiyeola and Bwapwa [6]. The mean values of the parameters measured are within the broad ranges reported in previous similar studies reported by Lemji and Eckstädt [42].

**Table 2:** Physicochemical characteristics of raw brewery wastewater in mg l^− 1^ except for temperature and pH

Parameter	Mean ± SD	Range
BOD_5_	1144 ± 431	667–1505
COD	2402 ± 1619	950–4149
NO_3_^−^ - N	11.27 ± 5.20	6.83–19
NH_4_^+^ − N	22.78 ± 3.91	15–34
SO_4_^−2^	15.33 ± 5.13	11–21
TKN	32.66 ± 6.81	25–38
TDS	2786 ± 960	1908–3811
TSS	2959 ± 123	2885 -
PO_4_^3−^-P	23.33 ± 8.33	14–30
T (°C)	31.33 ± 6.11	26–38
pH	6.36 ± 0.85	5.4–7

### Nutrients removal

#### Total Kjeldahl nitrogen (TKN)

**Fig. 2:**
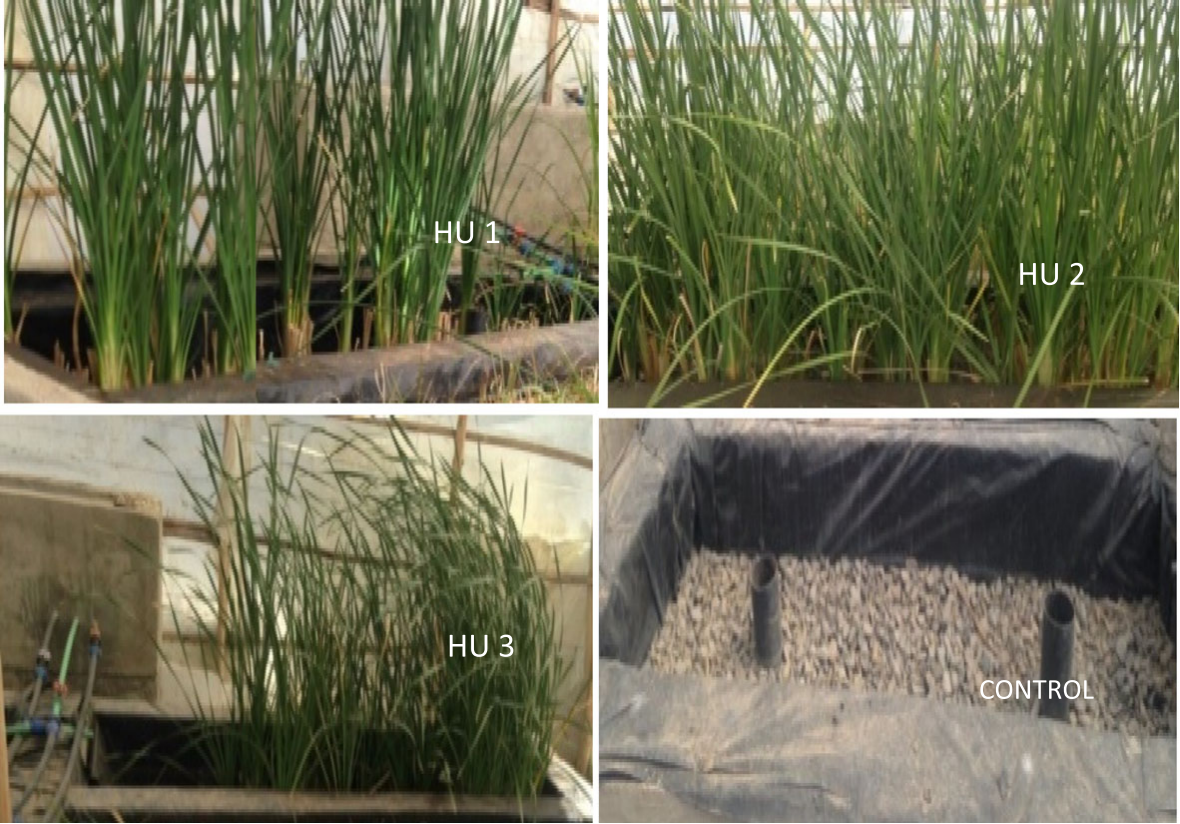
Triplicate hydroponic treatment units (HU1, HU2 and HU3) and control unit

The wastewater composition at the inlets of the HUs varied with time according to the beer brewing process (Table 4). Figure 2 shows reduction of nutrients by the hydroponic system planted with *T. latifolia*. The analysis of the results showed that TKN overall removal efficiencies were 69 and 41% for *Typha* planted and control units (for an inlet varying between 19 and 43 mg l^− 1^), respectively, reaching in some stages removal levels of up to 80% for the HUs. For this system the lower removal efficiency recorded was 54% (Table 3).

**Table 3:** Overall average (*±*SD) inlet and outlet concentrations (in mg l^− 1^ except for temperature and pH) and average percentage removal (%R) of wastewater constituents. Minimum and maximum values are indicated in brackets

Parameter	Inlet	Outlet			
		Control	%R	HUs	%R
TKN	33.29 ± 3.13 (19–43)	19.77 ± 1.87 (11–25)	41	10.14 ± 3.99 (5.38–17.67)	69
NH_4_^+^ − N	24 ± 1.63 (18–31)	18 ± 1.09 (14–23)	25	11.15 ± 0.69 (10.40–12.33)	54
NO_3_^−^ - N	13 ± 3.12 (8.70–17)	9.64 ± 2.52 (6.70–13)	26	6.4 ± 1.91 (4.30–9)	51
PO_4_^3−^ - P	14.86 ± 5 (8–23)	9.87 ± 2.57 (6.40–14)	34	6.26 ± 2.74 (3.44–10.80)	58
DO	0.83 ± 0.39 (0.3–1.4)	0.03 ± 0.05 (0–0.1)	–	0.02 ± 0.03 (0–0.07)	–
pH	6.4 ± 1.02 (4.8–7.8)	7.6 ± 0.64 (6.8–8.7)	–	8.1 ± 0.17 (8–8.3)	–
T(°C)	31.57 ± 4.72 (26–40)	20.29 ± 1.50 (18–22)	–	19.97 ± 0.86 (19.14–20.86)	–

Removal efficiencies of TKN were 69% (mean outlet concentration 10.26 ± 4.10 mg l^− 1^), 73% (mean outlet concentration 8.98 ± 5.33 mg l^− 1^) and 67% (mean outlet concentration 11.14 ± 4.85 mg l^− 1^) for the triplicate hydroponic treatment units.

#### Ammonium nitrogen (NH_4_^+^- N)

NH_4_^+^- N overall removal efficiencies were 54 and 25% for *Typha* planted and control units (for an inlet varying between 18 and 31 mg l^− 1^), respectively, reaching in some stages removal levels of up to 65% for the HUs. For this system the lower removal efficiency recorded was 42% (Table 4). For the triplicate hydroponic treatment units HU1, HU2 and HU3 the removal efficiencies of NH_4_^+^- N were 50% (mean outlet concentration 12 ± 1.79 mg l^− 1^), 58% (mean outlet concentration 9.9 ± 1.44 mg l^− 1^) and 52% (mean outlet concentration 11.54 ± 1.36 mg l^− 1^), respectively. Table 5 shows comparison of nutrient removal between the hydroponic and control units to indicate the role of Typha plants employed in the treatment.

**Table 4:** Inlet values and percentage removal (%R) of nutrients monitored on monthly basis for hydroponic treatment units (HUs) and control unit (CTRL)

Parameter	Description		Time (month)						
			23-May-15	23-Jun-15	23-Jul-15	23-Aug-15	23-Sep-15	23-Oct-15	23-Nov-15
TKN	Inlet (in mg l^−1^)		19	43	35	28	41	29	38
	Outlet	HUs (%R)	72	79	80	59	72	70	54
		CTRL (%R)	42	42	34	38	41	41	45
NH_4_^+^-N	Inlet (in mg l^−1^)		18	24	27	31	20	23	25
	Outlet	HUs (%R)	42	54	56	65	45	54	51
		CTRL (%R)	22	25	33	26	20	13	32
NO_3_^−^-N	Inlet (in mg l^−1^)		16	9.8	11.8	17	13	8.7	14.8
	Outlet	HUs (%R)	47	56	58	47	54	47	50
		CTRL (%R)	25	31	32	24	23	23	26
PO_4_^−3^-P	Inlet (in mg l^−1^)		13	11	16	14	8	19	23
	Outlet	HUs (%R)	59	56	63	70	57	51	53
		CTRL (%R)	23	27	40	37	20	35	39

**Table 5:** ANOVA and t-Test table for comparison of nutrient removal by the hydroponic treatment units planted with *T. latifolia* and the control unit in terms of statistical significance

ANOVA							
TKN							
Source of Variation		SS	df	MS	F	Interference	*P*-value
Between (Inlet and control) and (Inlet and HUs)		1892.84	2	946.4	25.34	S^a^	P < 0.001
Between (HU1 andHU2), (HU1 and HU3) and (HU2 and HU3)		16.6	2	8.3	0.36	–	*P* > 0.70
NH4 + -N							
Source of Variation		SS	df	MS	F	Interference	P-value
Between (Inlet and control) and (Inlet and HUs)		578.99	2	289.5	31.61	S	P < 0.001
Between (HU1 andHU2), (HU1 and HU3) and (HU2 and HU3)		17.08	2	8.54	3.59	–	P > 0.05
NO3--N							
Source of Variation		SS	df	MS	F	Interference	P-value
Between (Inlet and control) and (Inlet and HUs)		153.36	2	76.68	11.65	S	P < 0.001
Between (HU1 andHU2), (HU1 and HU3) and (HU2 and HU3)		6.06	2	3.03	0.61	–	P > 0.05
PO4–3-P							
Source of Variation		SS	df	MS	F	Interference	P-value
Between (Inlet and control) and (Inlet and HUs)		261.05	2	130.5	9.97	S	P < 0.001
Between (HU1 andHU2), (HU1 and HU3) and (HU2 and HU3)		4.51	2	2.26	0.29	–	P > 0.05
t-Test							
nutrients treated	TKN	NH4+ − N		NO3- - N		PO4–3- P	
Treatment units	CTRL HUS	CTRL HUS		CTRL HUS		CTRL	HUS
Mean	19.77 10.13	18 11.14		9.64 6.39		9.86	6.25
Variance	24.56 15.91	8.33 0.47		6.37 3.63		6.62	7.52
*P* value	P < 0.01	P < 0.05		P < 0.05		P < 0.05	
Interference	S	S		─		─	

S^a^ - significance

#### Nitrate (NO_3_^−^ - N)

The average removal efficiencies for NO_3_^−^-N of the treatment units were 51% for *Typha* planted units and 26% for the control (for an inlet varying between 8.7 and 17.0 mg l^− 1^), respectively, reaching in some stages removal levels of up to 58% for the HUs (Table 3). The maximum NO_3_^−^ -N removal efficiency 56% (outlet concentration 5.7 mg l^− 1^) was observed in the replicate hydroponic treatment unit HU2 followed by HU3 50% (outlet concentration 6.56 mg l^− 1^) and HU1 46% (outlet concentration 7.0 mg l^− 1^) Table 5.

#### Phosphorus (PO_4_^− 3^ - P)

The overall mean removal efficiencies of PO_4_^3−^- P were 58 and 34% for *T. latifolia* planted and control units (for an inlet varying between 8 and 23 mg l^− 1^) respectively (Table 3), reaching in some stages removal levels of up to 70% for the HUs) (Tables 3 and 4). The minimum removal efficiency observed by the system was 51%. Comparison of nutrient removal between hydroponic system and the control is shown in Table 5.

#### Plant growth analysis

The analysis identified the growth status of *T. latifolia* and changes in growth of the shoots as a result of wastewater feed in a continuous mode to the hydroponic treatment units (Table 6). The wastewater was supplied to HUs at a HLR of 2.3 cm d^− 1^ for HRT of 5 days. In all treatment units, the number of shoots increased and they appeared healthy. Growth of *Typha* was better in replicate hydroponic treatment unit HU2 than in units HU1 and HU3 (Table 6). The slight difference in number of plants per treatment units might be due to health condition of plants that may affect plant multiplication.

**Table 6:** Plant growth and nutrient analysis during the study period

Growth status	HUs		
	HU1	HU2	HU3
Initial number of *Typha* shoots	24	24	24
No.of plants at the end of the treatment	49	60	56
Height of plant (cm) at the end of the treatment period	110–190	100–175	115–170
Average no. of leaves per plant	5–9	6–10	5–11
No. of plants m^−2^	32	40	37
Dry biomass (kg) per unit	0.916	1.29	1.17
Dry biomass (kg m^−2^)	0.611	0.86	0.78
Accumulated nitrogen (g N kg^−1^ DW)	16.47	21.17	18.92
Accumulated phosphorus (g P kg^−1^ DW)	2.43	2.87	2.52

Sometimes wilting of shoots was also noticed, which might be due to variations of responses of individual plants to high levels of organic and/or nutrient loading of the influent that can cause stress to plants [43]. The increase in height of plants ranged from 1.0 m to 1.90 m and the number of leaves per plant ranged from 5 to 11 (Table 6) with little change during study period.

#### Plant biomass and nutrient content

Aboveground biomass of *T. latifolia* samples from each treatment unit were harvested from the gravel surface and assessed at the end of the experiment. The dry weight of biomass weighed and recorded for all treatment units is presented in Table 6. Values obtained were 0.61, 0.86, 0.78 kg dry DW m^− 2^ for replicate hydroponic treatment units HU1, HU2 and HU3, respectively. Nutrients accumulation in the samples harvested was found to be 16.47, 21.17 and 18.92 g N kg^− 1^ DW and 2.43, 2.87 and 2.52 g P kg^− 1^ DW for replicate hydroponic treatment unit HU1, HU2 and HU3, respectively, during the study period. Nutrients accumulation in the aboveground dry biomass per unit area were estimated from 10.06–18.21 g N m^− 2^ and 1.48–2.47 g P m^− 2^.

## Discussion

### Wastewater characteristics

In this study, the nutrients (TKN, NH_4_^+^-N, NO_3_^−^-N and PO_4_^3−^-P) values obtained exceeded the range of the effluent discharge standard of the country (80, 30 and 20 mg l^− 1^, respectively) [44]. The nutrient values were related to handling of raw material and amount of spent yeast present in the brewery effluent. Elevated phosphorus levels can also be the result of phosphorus containing chemicals used in the CIP units. The high values of nutrients in the wastewater imply that the effluent wastewater can cause pollution in the receiving water and other forms of environmental damage, especially in developing countries [31]. Thus, the wastewater has to be treated to reduce its environmental impact.

### Nutrients removal

The treatment of the wastewater using hydroponics planted *T. latifolia* revealed significant reductions of the nutrients. The outlet values of TKN were obviously lower than the inlet values (*p* < 0.05) during the monitoring period. In all the experiments, it was observed that TKN was reduced significantly (*p* < 0.001) in the HUs planted with *Typha* than in the control units (Table 3). ANOVA test indicated that the variation of removal by the replicate treatment units was insignificant (*p* > 0.05) (Table 5). Similarly, statistically different (*p* < 0.01) NH_4_^+^- N and NO_3_^−^-N reductions were observed in the HUs planted with *Typha* and the control units in all the experiments but they were insignificant (p > 0.05) among triplicate treatment units (HU1, HU2 and HU3) (Table 5).

It is expected that ammonia in the wastewater is the transformed organic nitrogen by the ammonification process due to the microbial activities. Nitrification process in the presence of oxygen again transformed the organic nitrogen to nitrite and then finally to nitrate. Ammonium is also up taken directly by plants in the hydroponics as nutrient and stored as organic nitrogen which further reduce its concentration from the wastewater. Further reduction of nitrogen content from the wastewater takes place by biomass assimilation in the microbiological processes which adsorbs NH_4_^+^-N.

The microbial nitrification process in the presence of oxygen advances in producing NO_3_^−^-N. While the anoxic conditions bring down amount of oxygen/air, high concentration of NO_3_^−^-N advances the microbial denitrification process. This brings about the change of NO_3_^−^-N into nitrogen gas, which escapes from the wastewater. As a result, NO_3_^−^-N get removed from the wastewater. Plants likewise add to the reduction by utilizing nitrate as nutrients which can be put away as natural nitrogen. This is on account of plants’ nutrient requirements which should be obtained from the wastewater instead of the gravel media. In the treatment units, the concentration of NO_3_^−^-N was small which may be because of anaerobic conditions that were not suitable for nitrification and good for denitrification of whatever NO_3_^−^-N was delivered in these units.

The overall mean removal rates achieved were 69, 54 and 51% for TKN, NH_4_^+^-N and NO_3_^−^-N, respectively by the hydroponic treatment units planted with *Typha* as compared to the corresponding 41, 25 and 26% removal of the control unit without *Typha*. The use of hydroponic system planted with *T. latifolia* improved the removal of nitrogen from 24 to 29% (Table 3) compared to the control unit. The outlet mean values of TKN, NH_4_^+^-N and NO_3_^−^-N from the hydroponic treatment system were within the range of the effluent discharge standard of the country (80, 30 and 20 mg l^− 1^, respectively) [44].

The finding of hydroponic treatment systems using *T. latifolia* with respect to nitrogen removal in this study is corroborated by previous studies of removal efficiencies 65, 70 and 80% [9, 21]*.* On the other hand, the removal efficiencies of our study (69, 54 and 51% for TKN, NH_4_^+^-N and NO_3_^−^-N, respectively) indicated better performance than in previous studies using free water surface constructed wetland planted with *Oenanthe javanica* [45] and subsurface flow constructed wetland planted with *Iris pseudacorus* (*I. pseudacorus*) [46]. Calheiros et al. [32, 47] reported reduction of 13–15% NO_3_^−^ - N, 18–42% TKN and 11–27% NH_3_ - N using constructed wetlands planted with mixture of *Canna indica, T. latifolia, Phragmites australis* and *I. pseudacorus* in treating tannery wastewater*.* They reported reductions of TKN similar to our study using two-stage constructed wetlands planted with *T. latifolia* and *P. australis* in treating industrial wastewater*.* Another study also reported lower removal efficiencies of nitrogen from wastewater using constructed wetlands planted with *Eichhornia crassipes*, *Lemnoideae* and *Pistia stratiotes* [48].

Removal of phosphorus fluctuated in line with the influent and other environmental factors which influenced the consequent removal efficiency similarly as nitrogen removal. The outlet values of phosphorus were significantly lower than their inlet values (*p* < 0.005) in all the experiments during the monitoring period. ANOVA test showed that the variation of removal by each replicate treatment unit was insignificant (*p* > 0.05) (Table 5). This might indicate the potential of establishing stable system for maintaining constant removal of phosphorus using hydroponics planted with *T. latifolia*. The removed amount of phosphorus in the HUs was higher than the amount left in the outlet. Statistically insignificant (*p* > 0.05) PO_4_^3−^- P reductions were observed between HUs planted with *Typha* and control units in all the experiments. But it is noted that the removal of phosphorus in the planted system was better than the unplanted system (control) (by 24%). This indicated role of plants in the removal of phosphorus, which may be due to direct uptake and microbial assimilation and by making favorable conditions for microorganisms to use phosphorus as a nutrient [33].

Phosphorus in wastewaters exists as phosphates in organic and inorganic forms. It is taken up by plant roots as phosphate, mainly as the predominant form of phosphate (PO_4_^3−^-P). Adsorption to filter media and detritus layer, precipitation and assimilation into microbial and plant biomass are the ways that PO_4_^3−^-P is removed from the wastewater. It is expected that the main removal mechanisms in sub-surface wastewater flow are adsorption and precipitation in the media matrix used [28, 49].

The removal of phosphorus increased at early stages and decreased at late stages of the experiment. This might be due to plant maturation and decreasing pore spaces of phosphorus adsorbing media of the treatment units because phosphorus uptake by macrophytes is usually highest during the beginning of the growing season [50]. Similar to nitrogen removal, 14 mg l^− 1^ of phosphorus concentration load in the inlet resulted in 4.23 mg l^− 1^ maximum removal (70%) in the outlet. This removal analysis revealed that as the concentration of phosphorus in the inlet wastewater deviated from 14 mg l^− 1^, the removal percentage of this nutrient decreased.

Removal of phosphorus tends not to be as high as nitrogen removal in wastewater treatment using macrophytes [51] because the macrophytes systems do not provide the direct metabolic pathway to remove phosphorus. Removal of phosphorus varied between 40 and 60% in most constructed wetlands was reported by Vymazal [50], which corroborates our results. Ayaz and Akca [52] reported phosphors removal efficiency of 48% in horizontal constructed wetland planted with *Cyperus*. For water hyacinth the average removal efficiencies for nitrogen were 40% and for phosphorus 18% [48].

Overall, the results revealed that hydroponic system with *T. latifolia* remove nutrients better than the control unit and also removed organic pollutants reported by [53] in the same experiment using this hydroponic treatment system. Removal of these nutrients could be attributed to plant and microbial uptake, and retention/adsorption onto gravel media. Contribution of the plants is direct removal of nutrients through both direct uptake and creation of conducive environment for microorganisms that use nitrogen and phosphorous as nutrients. The hydroponic systems also produce considerable biomass which can be utilized for different purposes. This synergy reinforces the potential use of *T. latifolia* as a value-added plant in wastewater treatment for removing nutrients.

Factors known to influence the removal mechanisms for nitrogen species in the wastewater are pH, temperature and dissolved oxygen (DO), including hydraulic characteristics such as water depth, HLR, and HRT [14, 20, 47]. This is because organisms present in biological wastewater treatments are sensitive to these factors. The range of optimum temperatures for nitrification and denitrification is between 16.5 and 32 °C and between 20 and 25 °C, respectively. Similarly, the range of pH favorable for nitrification and denitrification is between 6 and 9 [54]. The optimum range of pH is between 6.5 and 8.5, 8 and 9, and 7 and 9 for ammonification, nitrification and denitrification, respectively. A substantial drop in pH can hinder nitrification and denitrification [50].

In this study, wastewater inlet pH throughout the HUs operation varied between 4.80 and 7.80 and at the outlet ranged from 8.1 and 8.3 with a corresponding inlet temperature range of 26–40 °C and outlet range of 19.14–20.86 °C, both within the permissible limit (Table 3) [44]. The outlet pH reached 8.1 (which is slightly basic) after treatment units from an inlet value of 6.4. This is because microorganisms are consuming some organic acids in the process of bioremediation process. Thus, the ranges of pH and temperatures were within the normal range of operating conditions suitable for the pilot treatment system and were also optimum pH for *T. latifolia* development (3.0 and 8.5) [32].

The wastewater had high organic content which required high oxygen demand and resulted low level of DO at the inlet (mean value 0.83 mg l^− 1^) and outlet (mean value 0.02 mg l^− 1^). In addition, the reduction of DO might also be result of biological activity in root zone of the hydroponic bioreactor units. This is because DO is a source of energy for root respiration and growth. For aerobic removal of pollutants from the wastewater, oxygen might also be sourced from the atmosphere by diffusion into the *Typha* planted gravel medium and by continuous release of oxygen from the plant internal root zones in the rhizosphere [13]. A study showed that macrophytes used for wastewater treatments enhance root zone aeration [55]. In the ammonification of nitrogen removal process, the rates of ammonification increase in the oxygenated zone (near the roots and on the rhizosphere) and then decrease in the anaerobic zones. The nitrification process requires oxygen and is sensitive to DO levels. Removal of nitrogen from wastewater into gaseous compounds takes place by the processes of nitrification and denitrification since nitrogen is usually found in a reduced state in the wastewater. But rates of denitrification are determined by slower nitrification rates which implies that both aerobic and anaerobic conditions are required for the process of denitrification in the removal of nitrogen.

Gravel alone reduced the amount of input nitrogen between the inlet and outlet control unit by 48%, indicating its contribution in wastewater treatment. The ability of gravel alone to remove nitrogen might be related to the ability of binding sites on the gravel and formation of a microbial film on the surface of the gravel [27]. The use of *Typha* (planted vs. unplanted units) further improved the removal efficiency significantly (*p* < 0.05). The improvements can be attributed to direct nutrient uptake by the plants for growth, and to the actions of microbes harbored in the rhizosphere [33, 43].

Better nutrient removal may also be due to suitable wastewater composition of the influent for the treatment system although other factors, including individual health conditions of the plants, pH, and ambient temperature may have contributed to the outcome. Under the given conditions of this study, 35 mg l^− 1^ of TKN concentration load in the inlet resulted maximum removal (80%) in the outlet. The result of this removal analysis revealed that as the concentration of TKN in the inlet wastewater deviated from 35 mg l^− 1^, the removal percentage of the nutrient decreased (Table 4). Similarly, maximum removal of NH_4_^+^- N (65%) and NO_3_^−^- N (58%) was achieved for inlet concentrations of 31.0 and 11.8 mg l^− 1^, respectively. Removal of nutrients could also vary with patterns of plant growth, and the most vigorous growth period corresponded with high nutrient removal rates [13, 50]. Minimum removal could be related to plant senescence, which might indicate plant harvesting to replenish for continuous and steady treatment [56]. Thus the combined action of microbes, plant uptake and retention/adsorption onto gravel media has resulted in better removal of nitrogen in the hydroponic bioreactor treatment units compared to the control.

Similar to removal impacts of nitrogen, phosphorus removal from wastewater is also influenced by the pH in the water. Due to acidic nature of the wastewater at low pH (< 5), it is difficult for macrophytes to perform the removal. Similarly, high pH value (> 9) of the wastewater impedes performance of the macrophytes. A study indicated that pH ranges of wastewater from 6 to 9 is good condition for the performance of macrophytes in the removal of nutrients [54]. The pH range of the wastewater in this study is within the suitable range (8–8.3) (Table 3). It is also reported that temperatures below 15 °C are not suitable for microorganisms and plants that can contribute to the removal of the nutrients [28]. Hence phosphorus removal is temperature dependent, and in the present study the average temperature was in the suitable range (19 - 21 °C) (Table 3). It is also reported that aerobic conditions are favorable for P sorption and precipitation [57].

In summary, it is noted that hydroponic technology using *Typha latifolia* is potentially capable of removing nutrients from brewery wastewater. The removal of nutrients takes place in the rhizosphere of the plants which favors aerobic nitrifying bacteria by providing oxygen through roots of the plant from the atmosphere, root surface area for attached growth and release of root exudates as energy source [33]. The reduction of nutrients is believed to be carried out by the combination of physical, chemical and biological processes. Among these processes sedimentation, filtration, biological degradation, adsorption and nutrient uptake could enhance the removal from the wastewater [15].

#### Plant growth analysis

*T. latifolia* became established and grew well under real exposure of brewery wastewater loads and showed good capacity to remove nutrients in greenhouse conditions. This was due to provision of nutrients, water and support media from the designed hydroponic system [25].

The three HUs provided a good platform for *T. latifolia* establishment and were suited for the intended nutrient removal purpose. This could be due to the fact that they were facing similar wastewater load and the same environmental conditions. Density of plants near the inlets of the HUs, where pollutant loading could be higher, decreased because of more wilting and some mortality of plants than in the middle of the tanks and near the outlets.

In addition, plants located nearer to outlets were thick green, robust and taller than plants nearer to inlet in all treatment units. This could be due to decreased pollutant loading moving down to the outlets of treatment units and associated decreased stress on plants. However, differences in plant growth among treatments units were small (Table 6), indicating that *T. latifolia* was reacting similarly to the imposed conditions in each replicate unit. Other research involving this species showed successful establishment and plant growth in constructed wetlands [32].

#### Plant biomass and nutrient content

The results of dry biomass for the *T. latifolia obtained* were in agreement with the average aboveground biomass range (0.3–1.8 kg DW m^− 2^) that was reported by Maddison et al. [30]. Other studies reported similar aboveground biomass ranged from 0.21–0.85 kg DW m^− 2^ and exceeding 0.5 kg DW m^− 2^ of *T. latifolia* employed in constructed wetlands for wastewater treatment [33, 58, 59]. On the other hand, the values obtained in the present study are lower than the aboveground biomass of *T. latifolia* reported by Solano et al. [60] and Toet et al. [61]. Relatively more aboveground biomass was recorded (0.86 kg DW m^− 2^) for replicate hydroponic treatment unit 2. This could be due to higher plant density (40 plant m^− 2^) (Table 6). Accordingly, the estimated nutrient accumulations in the aboveground dry biomass were ranged from 15.10–27.31 g N and 2.23–3.70 g P in the replicate treatment units. Tanner [38] reported above-ground N and P concentrations ranging from 15 to 32 g N kg^− 1^ DW and 1.3 to 3.4 g P kg^− 1^ DW, which is in agreement with the present study. Replicate hydroponic treatment unit 2 accumulated slightly more nutrients (21.17 g N kg^− 1^ DW and 2.87 g P kg^− 1^ DW).

As can be seen from the results, nutrients accumulated in plants were proportional to biomass produced. This was also related to pollutant removal from the wastewater. Thus, the ability of plants to decrease nutrients in wastewater as a function of nutrient uptake and biomass production (biomass of *T. latifolia* is suitable for energy) [60] plays an important role in wastewater treatment. It is important to note that nutrient content of wastewater is a valuable resource when utilized properly for reuse, whereas untreated discharge to water bodies can cause eutrophication resulting in ecological damage [49].

## Conclusion

Hydroponics technology using *T. latifolia* were designed, constructed and operated to evaluate its suitability for brewery wastewater treatment. *T. latifolia* became established and grew well under real exposure of brewery wastewater loads and showed good capacity to remove nutrients in greenhouse conditions. The hydroponics technology was efficient in removing nutrients with removal efficiencies of 69, 54, 51 and 58% for TKN, NH_4_^+^-N, NO_3_^−^-N and PO_4_^3−^-P, respectively under the given conditions and produced considerable biomass. These findings reveal that hydroponics technology is a promising ecological option for wastewater treatment. Further research to improve integration of wastewater treatment and biomass production is required. Based on wastewater characteristics, it is important to develop alternative methods that can integrate removal of nutrients and production of valuable biomass using biological processes. The establishment of a research consortium that addresses the problem of agro-food processing industries wastewater, including breweries, its remediation and reuse by selecting relevant techniques such as biological nutrient removal for reduction in nitrogen and phosphorus may promote and guide these efforts. In addition, it is needed to investigate and explore vital role played by microorganisms in application of hydroponics using macrophytes for wastewater treatment.

## Abbreviations

AAU: Addis Ababa University
ANOVA: One-way analysis of variance
APHA: American Public Health Association
BOD_5_: 5 days biological chemical demand
CIP: Cleaning in place
COD: Chemical oxygen demand
CTRL: Control
DO: Dissolved oxygen
DW: Dry weight
EPA: Ethiopian Environmental Protection Authority
GDP: Gross domestic product
HED: Higher Education Development
HLR: Hydraulic loading rate
HRT: Hydraulic retention time
HU1: Hydroponic treatment unit 1
HU2: Hydroponic treatment unit 2
HU3: Hydroponic treatment unit 3
HUs: Hydroponic bioreactor treatment units
SD: Standard deviation
SPSS: Statistical Product and Service Solutions
TDS: Total dissolved solids
TKN: Total Kjldahal Nitrogen
TP: Total phosphorus
TSS: Total suspended solids
*Typha latifolia*: *T. latifolia*
USAID: United States Agency for International Development
V_gravel_: Volume of gravel
V_void_: Void volume

## References

[1] Van Rooijen DJ, Biggs TW, Smout I, Drechsel P (2010). Urban growth, wastewater production and use in irrigated agriculture: a comparative study of Accra, Addis Ababa and Hyderabad. Irrig Drain Systems.

[2] Gebre G, Rooijen D. Urban water pollution and irrigated vegetable farming in Addis Ababa. In: Water, sanitation and hygiene: Sustainable development and multisectorial approaches. Proceedings of the 34th WEDC International Conference. Addis Ababa: United Nations Confrence Centre; 2009. p. 18–22.

[3] WWAP (2017). The United Nations world water development report 2017: Wastewater. The Untapped Resource.

[4] Rao KR, Hailu FK (2016). Environmental corporate social responsibility of brewery firms in Ethiopia. IJAR.

[5] Brewers of Europe. Guidance note for establishing BAT in the brewing industry. Brussels; 2002.

[6] Jaiyeola AT, Bwapwa JK (2016). Treatment technology for brewery wastewater in a water-scarce country: a review. S Afr J Sci.

[7] Aschale M, Sileshi Y, Kelly-Quinn M, Hailu D (2015). Potentially toxic trace element contamination of the little Akaki River of Addis Ababa. Ethiopia J Nat Sci Res.

[8] Trivedy R (2007). Low cost and energy saving technologies for water and wastewater treatment. Control Pollution.

[9] Renuka N, Sood A, Prasanna R, Ahluwalia A (2015). Phytoremediation of wastewaters: a synergistic approach using microalgae for bioremediation and biomass generation. Int J Environ Sci Technol.

[10] Keeratiurai P (2013). Efficiency of wastewater treatment with hydroponics. ARPN J Agr Biol Sci.

[11] Bawiec A, Pawęska K, Pulikowski K (2016). Seasonal changes in the reduction of biogenic compounds in wastewater treatment plants based on hydroponic technology. J Ecol Eng.

[12] Abe K, Kato K, Ozaki Y (2010). Vegetation-based wastewater treatment technologies for rural areas in Japan. JARQ.

[13] Stottmeister U, Wießner A, Kuschk P, Kappelmeyer U, Kästner M, Bederski O, Müller RA, Moormann H (2003). Effects of plants and microorganisms in constructed wetlands for wastewater treatment. Biotechnol Adv.

[14] Saeed T, Sun G (2012). A review on nitrogen and organics removal mechanisms in subsurface flow constructed wetlands: dependency on environmental parameters, operating conditions and supporting media. J Environ Manag.

[15] Vinita V, Singh U, Billore S (2008). Efficiency of root zone technology for treatment of domestic wastewater. Proceedings of the 12th international world lake conference.

[16] Vymazal J, Kropfelova L. Wastewater treatment in constructed wetlands with horizontal subsurface flow. In: Environmental pollution, Alloway B, Trevors J, (Eds), Springer, Czech Republic. 2008;14:11–45.

[17] López-Chuken UJ, Asaso T (2012). Hydroponics and environmental clean-up. Hydroponics – a standard methodology for plant biological researches.

[18] Dipu S, Anju A, Kumar V, Thanga SG (2010). Phytoremediation of dairy effluent by constructed wetland technology using wetland macrophytes. Global J Environ Res.

[19] Saeed T, Sun G. A comprehensive review on nutrients and organics removal from different wastewaters employing subsurface flow constructed wetlands. Critical Reviews in Environ Sci Technol. 2017; 00-00. (just-accepted)

[20] Osem Y, Chen Y, Levinson D, Hadar Y (2006). The effects of plant roots on microbial community structure in aerated wastewater-treatment reactors. Ecol Eng.

[21] Shalaby I, Altalhy A, Mosallam HA (2008). Preliminary field study of a model plant for sewage water treatment using gravel bed hydroponics method. World Appl Sci J.

[22] Haddad M, Mizyed N, Masoud M (2012). Evaluation of gradual hydroponic system for decentralized wastewater treatment and reuse in rural areas of Palestine. Int J Agr Biol Eng.

[23] Mant C, John P, Eric M, John B (2003). A feasibility study of a *Salix viminalis* gravel hydroponic system to renovate primary settled wastewater. Bioresour Technol.

[24] Norstorm A. Treatment of domestic wastewater using microbiological processes and hydroponics in Sweden. Ph.D, thesis; Royal Institute of Technology, Stockholm, Sweden. 2005;91-7178-030-0.

[25] Mavrogianopoulos G, Vogli V, Kyritsis S (2002). Use of wastewater as a nutrient solution in a closed gravel hydroponic culture of giant reed. Bioresour Technol.

[26] Snow A, Ghaly AE (2008). A comparative assessment of hydroponically grown cereal crops for the purification of aquaculture waste water and the production of fish feed. Am J Agr Biol Sci.

[27] Qomariyah S, Ramelan A, Setyono P (2017). Use of macrophyte plants, sand & gravel materials in constructed wetlands for greywater treatment. IOP Conference Series: Materials Science and Engineering.

[28] Akratos CS, Tsihrintzis VA (2007). Effect of temperature, HRT, vegetation and porous media on removal efficiency of pilot-scale horizontal subsurface flow constructed wetlands. Ecol Eng.

[29] Wu H, Zhang J, Ngo HH, Guo W, Hu Z, Liang S, Fan J, Liu HA (2015). A review on the sustainability of constructed wetlands for wastewater treatment: design and operation. Bioresour Technol.

[30] Maddison M, Mauring T, Remm K, Lesta M, Mander Ü (2009). Dynamics of *Typha latifolia L.* populations in treatment wetlands in Estonia. Ecol Eng.

[31] Aslam MM, Hassan S, Baig M (2010). Removal of metals from the refinery wastewater through vertical flow constructed wetlands. Int J Agri Biol.

[32] Calheiros CS, Rangel AO, Castro PM (2007). Constructed wetland systems vegetated with different plants applied to the treatment of tannery wastewater. Water Res.

[33] Vymazal J (2011). Plants used in constructed wetlands with horizontal subsurface flow: a review. Hydrobiologia.

[34] Lishenga IW, Nyaanga DM, Owino JO, Wambua RM (2015). Efficacy of hydroponic and soil-based vetiver Systems in the Treatment of domestic wastewater. Int J Pure Appl Sci Technol.

[35] Pastor R, Benqlilou C, Paz D, Cardenas G, Espuña A, Puigjaner L (2003). Design optimisation of constructed wetlands for wastewater treatment. Resour Conserv Recy.

[36] Kantawanichkul S, Kladprasert S, Brix H (2009). Treatment of high-strength wastewater in tropical vertical flow constructed wetlands planted with *Typha angustifolia* and *Cyperus* involucratus. Ecol Eng.

[37] APHA (1999). Standard Methods for the Examination of Water and Wastewater, 20th edn.

[38] Tanner Chris C. (1996). Plants for constructed wetland treatment systems — A comparison of the growth and nutrient uptake of eight emergent species. Ecological Engineering.

[39] Brisson J, Chazarenc F (2009). Maximizing pollutant removal in constructed wetlands: should we pay more attention to macrophyte species selection?. Sci Total Environ.

[40] Zhang Z, Rengel Z, Meney K (2007). Removal of nutrients from secondary-treated municipal wastewater in wetland microcosms using ornamental plant species. Int J Environ Waste Manag.

[41] Chen Y, Bracy RP, Owings AD, Merhaut DJ (2009). Nitrogen and phosphorous removal by ornamental and wetland plants in a greenhouse recirculation research system. Hortscience.

[42] Lemji HH, Eckstädt H (2015). Efficiency of a pilot scale trickling filter to treat industrial brewery wastewater: influence of hydraulic loading. J Chem Technol Biotechnol.

[43] Environmental Protection Authority (EPA). Standards for industrial pollution control in Ethiopia. Prepared by the Federal Environmental Protection Authority and the United Nations Industrial Development Organization Under the ecologically sustainable industrial development, Addis Ababa, Ethiopia, 2003.

[44] Zheng S, Yang Z, Sun M (2010). Pollutant removal from municipal sewage in winter via a modified free-water-surface system planted with edible vegetable. Desalination.

[45] Youssef Z, Mohseni-Bandpei A (2010). Nitrogen and phosphorus removal from wastewater by subsurface wetlands planted with *iris pseudacorus*. Ecol Eng.

[46] Calheiros CS, Rangel AO, Castro PM (2009). Treatment of industrial wastewater with two-stage constructed wetlands planted with *Typha latifolia* and *Phragmites australis*. Bioresour Technol.

[47] Shah M, Hashmi HN, Ghumman AR, Zeeshan M (2015). Performance assessment of aquatic macrophytes for treatment of municipal wastewater. J S Afr Institution of Civil Eng.

[48] Vymazal J (2010). Constructed Wetlands for Wastewater Treatment. Review Water.

[49] Vymazal J (2007). Removal of nutrients in various types of constructed wetlands. Sci Total Environ.

[50] Brix H (1994). Functions of macrophytes in constructed wetlands. Water Sci Technol.

[51] Ayaz SC, Akça L (2001). Treatment of wastewater by natural systems. Environ Int.

[52] Abebe W, Nurelegne T, Helmut K, Solomon B. Constructed wetlands for phytoremediation of industrial wastewater in Addis Ababa, Ethiopia. Nanotechnol Environ Eng. 2018. 10.1007/s41204-018-0038-y.

[53] Shah Mumtaz, Hashmi Hashim, Ali Arshad, Ghumman Abdul (2014). Performance assessment of aquatic macrophytes for treatment of municipal wastewater. Journal of Environmental Health Science and Engineering.

[54] Brix H, Schierup HH, Cooper PF (1999). Soil oxygenation in constructed reed beds: The role of macrophytes and soil-atmosphere interface oxygen transport. Constructed Wetlands in Water Pollution Control.

[55] Bindu T, Sylas VP, Mahesh M, Rakesh PS, Ramasamy EV (2008). Pollutant removal from domestic wastewater with Taro *(Colocasia esculenta*) planted in a subsurface flow system. Ecol Eng.

[56] Yang Z, Wang Q, Zhang J, Xie H, Feng S (2016). Effect of plant harvesting on the performance of constructed wetlands during summer. Water.

[57] Vymazal J (2005). Horizontal sub-surface flow and hybrid constructed wetlands systems for wastewater treatment. Ecol Eng.

[58] Wang L, Gan H, Wang F, Sun X, Zhu Q (2010). Characteristic analysis of plants for the removal of nutrients from a constructed wetland using reclaimed water. CLEAN–Soil Air Water.

[59] Salem ZB, Laffray X, Al-Ashoor A, Ayadi H, Aleya L (2017). Metals and metalloid bioconcentrations in the tissues of *Typha latifolia* grown in the four interconnected ponds of a domestic landfill site. J Environ Sci.

[60] Solano M, Soriano P, Ciria M (2004). Constructed wetlands as a sustainable solution for wastewater treatment in small villages. Biosyst Eng.

[61] Toet S, Bouwman M, Cevaal A, Verhoeven JT (2005). Nutrient removal through autumn harvest of *Phragmites australis* and *Typha latifolia* shoots in relation to nutrient loading in a wetland system used for polishing sewage treatment plant effluent. J Environ Sci Health.

